# The Relationship of Alcohol to ART Adherence Among Black MSM in the U.S.: Is it Any Different Among Black MSM in the South?

**DOI:** 10.1007/s10461-021-03479-3

**Published:** 2021-11-06

**Authors:** Shantrel S. Canidate, Eric W. Schrimshaw, Nancy Schaefer, Nioud Mulugeta Gebru, Noelani Powers, Stephen Maisto, Christina Parisi, Robert F. Leeman, Sheldon Fields, Robert L. Cook

**Affiliations:** 1 Department of Epidemiology, College of Public Health and Health Professions and College of Medicine, University of Florida, 2004 Mowry Road, PO Box 100231, Gainesville, FL 32610-0182 USA; 2 Department of Population Health Sciences, College of Medicine, University of Central Florida, Orlando, FL 32827 USA; 3 UF Health Science Center Libraries, University of Florida, Gainesville, FL 32610 USA; 4 Department of Health Education and Behavior, College of Health and Human Performance, University of Florida, Gainesville, FL 32611 USA; 5 Department of Psychiatry, College of Arts and Sciences, Syracuse University, Syracuse, NY 13244 USA; 6 Department of Psychiatry, Yale School of Medicine, Yale University, New Haven, CT 06511 USA; 7 College of Nursing, The Pennsylvania State University, University Park, PA 16802 USA

**Keywords:** Alcohol, ART adherence, Black men who have sex with men, United States, Southern US

## Abstract

Alcohol-using Black MSM (Men who have sex with men) are disproportionately impacted by HIV in the U.S.—particularly in the southern U.S.—despite the availability of antiretroviral therapy (ART). The purpose of this study was to summarize the current evidence on alcohol use and ART adherence among Black MSM in the U.S. and in the South and to identify future research needs. A systematic review was conducted using eight databases to identify relevant peer-reviewed articles published between January 2010 and April 2021. The authors also snowballed remaining studies and hand-searched for additional studies. Including both quantitative and qualitative studies, five published studies examined alcohol and ART adherence among Black MSM in the U.S. The search identified 240 articles, the study team reviewed 114 in full-text and determined that only five met the inclusion criteria. Three of the five included studies identified alcohol use as a barrier to ART adherence. In conclusions, the general lack of literature on HIV disparities among alcohol-using Black MSM in the U.S. (specifically in the South) indicates a critical need for research on this population’s unique risks and needs to inform the development of tailored interventions.

**Supplementary Information:**

The online version contains supplementary material available at 10.1007/s10461-021-03479-3.

## Introduction

Black/African American (referred hereafter as Black) men who have sex with men (MSM) in the United States (U.S.) experience HIV-related disparities that may directly or indirectly be exacerbated by alcohol use. This is particularly the case in the southern US. Despite the effectiveness of treatment as prevention approaches (TasP) such as antiretroviral therapy (ART) in reducing the transmission of HIV, substantial evidence suggests that Black MSM in the U.S. are less likely to adhere optimally to ART compared to other MSM [1–3]. One documented factor associated with ART nonadherence among people with HIV (PWH) is heavy alcohol use [4]. While the rates of alcohol use among MSM in the U.S. are high, only a little week evidence suggests that rates of alcohol use are higher among Black MSM—including those in the South—than among their counterparts [5–8]. Thus, the relationship of alcohol to ART adherence could be different in this population. Therefore, the goals of the paper were to summarize the current evidence on alcohol use and ART adherence among Black MSM in the South and to identify future research needs. Following a brief overview of differences in HIV-related outcomes among Black MSM in the U.S. and in the South, we provide findings from our systematic review, then discuss recommended next steps.

### HIV Incidence and Prevalence Rates Among Black MSM in the U.S.

HIV incidence and prevalence indicate racial and ethnic disparities among Black MSM in the U.S. compared to other groups [9, 10]. A prior review used meta-analytic methods to calculate overall HIV incidence and projected HIV prevalence over time using a simulated cohort of Black MSM aged 18–40 in the U.S. [9]. The authors determined that a 4.16% incidence rate would lead to a projected HIV prevalence of 61% by age 40 among Black MSM in the U.S. Another study examined changes in disparities in estimated HIV incidence rates among Black and other MSM in the U.S. using data collected from 2010 to 2015 from the Centers for Disease Control and Prevention (CDC) Supplemental Surveillance Report [10]. The authors reported increasing HIV incidence in Black MSM from 2010 and 2015 in Black MSM but no change in rate(s?) of disparities between Black and White MSM [10]. Therefore, addressing racial and ethnic disparities in the U.S may greatly reduce overall annual HIV incidence and prevalence among Black MSM.

### HIV Incidence and Prevalence Rates Among Black MSM in the South

HIV infection is particularly common in the southern U.S., commonly referred to as the South. The South includes Alabama, Arkansas, Delaware, Florida, Georgia, Kentucky, Louisiana, Maryland, Mississippi, North Carolina, Oklahoma, South Carolina, Tennessee, Texas, Virginia, West Virginia, and Washington, District of Columbia (D.C.) [10–18]. In 2017, the South was home to only 38% of the U.S. population [10, 16] but had in excess of 50% of the new HIV diagnoses in the country [14, 15]. The nine states commonly and collectively known as the Deep South (Alabama, Florida, Georgia, Louisiana, Mississippi, North Carolina, South Carolina, and Texas) were disproportionately impacted by HIV disease [14, 17].

In 2017, the South housed more than 50% of HIV diagnoses, 45% of persons with HIV, and 50% of undiagnosed infections [10, 16, 17]. In the Deep South, HIV diagnosis rates were higher among Black people in 2014 (54%) than the national average overall in the US (44%). [14, 17]. Furthermore, new HIV diagnoses in Black and Latino MSM in the Deep South increased 67% in 2014 [14]. Likewise, in 2017, 81% of the new HIV diagnoses in the Deep South were attributed to MSM, with notable increases among Black and Latino populations since 2014 [10, 14, 16, 17]. Understanding the factors contributing to disparities in HIV outcomes among Black people—especially Black MSM in the South—will be crucial in designing measures to reduce or eliminate these racial and geographic disparities.

### Disparities in HIV Outcomes Among Black MSM in the U.S.

In examining the disparities in HIV outcomes between Black and other racial/ethnic MSM in the U.S., previous reviews have reported inconsistent findings. Although the evidence is mostly observational and sometimes contradictory, the reviews have generally concluded that Black MSM tend to have higher rates of sexually transmitted infections (STIs), lower adherence to ART, delayed HIV diagnosis, and less uptake of HIV testing than White MSM [1, 3, 19]. Disparities in rates of HIV infection between Black MSM and their counterparts could not be explained entirely by engagement in high-risk sexual behavior or reported use of substances (i.e., alcohol or illicit drugs). Two published meta-analyses by Millet et al. [2, 20] examined disparities and risks of HIV infection in Black and White MSM in the U.S. and abroad. The authors of the 2007 analysis found that Black MSM reported less overall substance use and lower adherence to ART [2]. The authors of the 2012 analysis found that Black MSM in the U.S. were less likely to have health insurance, report a history of substance use, adhere to ART, or be virally suppressed compared to other HIV-positive MSM in the U.S. [20]. While most of these findings emphasized individual behaviors and individual biological factors such as STIs, the reviews and meta-analyses also highlighted the potential contribution of social and sexual networks, and socio-cultural factors at the community and policy levels.

### Disparities in HIV Outcomes Among Black MSM in the South

In addition to individual factors such as alcohol use, documented structural and social factors that are prevalent in the South contribute to HIV-related disparities. These factors include institutional racism, lack of trust in the health care system and government, lack of state and federal funding for prevention services, and higher rates of poverty, incarceration, STIs and HIV-related stigma [10, 14, 16, 17]. Higher rates of STIs, homophobia, and HIV-related stigma have been reported among Black MSM in the South and have been associated with facilitating HIV transmission and limiting access to HIV prevention services [2, 3, 16]. In addition, Black MSM in the South who experience multiple stigmas [22], in response to their race, sexuality, and HIV status, may be more likely to use alcohol or drugs. Furthermore, Black MSM may experience greater adverse effects from the same level of consumption as White MSM. Because while White MSM have reported more drinking, Black MSM may engage in drinking while also experiencing structural and social factors including poverty and racism.

### Alcohol Use and ART Adherence

To date, Black MSM in the South continue to have the least favorable HIV care continuum (HCC) outcomes including delayed ART initiation, short retention in care, and long time to achieving viral suppression [15]. The HIV care continuum outlines the steps that persons living with HIV (PLWH) take from diagnosis to achieving and maintaining viral suppression [18, 23, 24]. It provides key potential for improving outcomes in the U.S. and in the South, as well as reducing HIV-related disparities among high-risk populations such as Black MSM [18, 23, 24]. ART adherence is one of the most important but often difficult routes to HIV clinical outcomes and prevention in PLWH. Creating and implementing HIV-related initiatives that are both relevant and culturally appropriate and that reach the groups most affected by HIV can be challenging. Despite ART’s great success as a prevention method against the development of AIDS or HIV-related deaths, adherence to ART medication is often poor [4, 25–31]. Treatment adherence (i.e., taking medication as prescribed) is generally regarded by healthcare professionals as the most important factor in HIV treatment success: nonadherence can contribute to treatment failure and disease progression [4]. When taken consistently and correctly, ART reduces both the amount of HIV in the body and the onward transmission of HIV [4, 32]. Recent changes in the guidelines on the use of ART in PWH indicate that at least 80–90% adherence is now considered necessary for viral suppression [4].

Substance use is often associated with poor HIV-related outcomes and an increased risk of HIV transmission [33–35]. Substance use is prevalent among PLWH, cited as one of the correlates of ART nonadherence and associated with medication nonadherence in studies such as Woolf-King et al. and Hendershot et al. [4, 25]. While much of the literature has focused on the association between injection drug use (IDU) and ART adherence, the volume of literature focusing on alcohol has begun to increase [4, 25–31]. However, the nature, strength, and consistency of the association between alcohol and ART adherence remain unclear [4, 25, 27]. Previous reviews and meta-analyses examining the association of alcohol consumption with ART adherence have also yielded conflicting results [27, 28]. Reasons for the discrepancies include methodological differences in measuring alcohol use [28], failure to examine different patterns of alcohol use on HIV medication adherence [27], and the lack of a theoretical model to better understand the mechanisms by which alcohol influences ART adherence [4]. To address the latter, an integrated conceptual model of alcohol consumption and ART adherence (i.e., the Information-Motivation-Behavioral Skills (IBM) model and the theory on the acute effects of alcohol ingestion) has been proposed to describe the causal effect of alcohol consumption on ART nonadherence [4]. However, the theory does not address race and geographic region, thus resulting in the need for additional research that factors in both of these additional factors.

### Current Alcohol-Related Interventions for Black MSM Living with HIV

The previous sections have shown that considerable research has focused on examining individual-level effects of alcohol use on HIV incidence or HIV-related outcomes [36–40]. As discussed, documented social and cultural factors have been reported among Black MSM in the South and may directly or indirectly influence the relationship between alcohol and HIV transmission. Alcohol is not a major focus of any of the current recommended HIV-prevention interventions for Black MSM in the U.S. or in the South. Addressing this gap may well require an explanation of how alcohol contributes to HIV transmission risk in this population through unique pathways at the individual (biological, behavioral), network, community, or policy levels.

The Centers for Disease Control and Prevention (CDC) has published a compendium of evidence-based interventions and best practices for HIV prevention [41]. The majority of the extant evidence-based behavioral interventions focus on risk reduction practices, including consistent and correct condom use as well as partner communication [16]. In recent years, the introduction of biomedical interventions including ART adherence/uptake has resulted in a paradigm shift in HIV prevention strategies [15]. Unfortunately, these prevention strategies have been less effective in eliminating HIV-related disparities in the South [15]. The South continues to account for 50% of all diagnoses of HIV infection among MSM [10, 16, 17]. Regarding tailored interventions for Black MSM, the CDC compendium lists six interventions proposed by four studies [42–45]. The interventions target different goals across the HIV care continuum, including (1) improving retention in HIV care and ART adherence (i.e., Project nGage and Strength Through Livin’ Empowered [STYLE]); (2) increasing PrEP initiation/uptake (i.e., PrEP counseling center), and (3) reducing risky sexual behaviors (Many Men, Many Voices [3MV]) [42–45].

Most of these interventions apply at the individual level, although one includes a structural-level intervention (increasing access to HIV testing by using non-traditional testing sites such as venue-based and social/sexual network testing). Only one of these proven and recommended interventions addresses alcohol or substance misuse, yet alcohol is a modifiable behavior, offering the possibility of improving disease management and delaying rapid disease progression [25]. HIV preventive interventions could benefit Black MSM in the South by incorporating alcohol-related behavioral research into event-specific contexts that link alcohol consumption to risky behavior. Interventions that have proven effective at changing individual drinking behavior may need to be culturally adapted for this population. Identifying what is known about the extent to which alcohol is contributing to the ongoing risk of HIV transmission and health disparities among Black MSM in the South holds the promise of addressing these problems.

### Purpose of this Review

We found no systematic review of the literature examining the association between alcohol use and ART adherence among Black MSM in the U.S. overall nor specifically in the South. The aims of this study were to (1) systematically review the literature on alcohol use and ART adherence among Black MSM in the U.S. and in the South in order to summarize the current state of knowledge on this issue; (2) describe the characteristics of studies that examined this association; (3) summarize the findings; and (4) provide recommendations for future research related to alcohol use interventions and the prevention of HIV.

## Methods

### Search Strategy

**Fig. 1:**
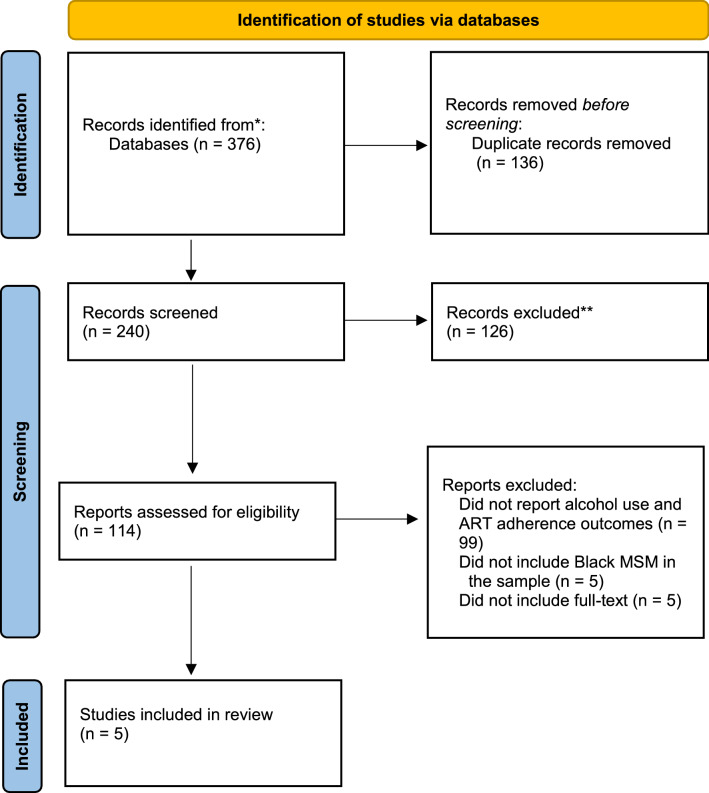
PRISMA flowchart of study selection process

A health sciences librarian (NS) searched eight databases (PubMed, Embase, Cochrane Library, Web of Science, International Pharmaceutical Abstracts and Ebscohost CINAHL, APA PsycINFO, and Academic Search Premier) for studies published in English from January 2010 until April 2021 (see Fig. 1 for the Preferred Reporting Items for Systematic Reviews and Meta-Analyses [PRISMA] [46, 47] flowchart of systematic reviews (Fig. 1). The search strategy used keywords for four concepts: (1) Black/African American, (2) men who have sex with men (MSM [including homosexual, gay, bisexual, transgender, queer (GBTQ)]), (3) HIV/AIDS, and (4) medication (non) adherence/compliance. The full list of words used are attached as a supplement.

### Study Selection

The 376 results from the electronic searches were imported into COVIDENCE [48] and de-duplicated. Titles and abstracts of the resulting 240 unique studies were initially screened by the first author (SC) and a co-author (NP) to exclude studies that were not relevant. A total of 114 met preliminary inclusion criteria for full-text review. The following preliminary inclusion criteria were applied: (1) published in English, (2) published between 2010 and 2021, (3) needed to be full-text manuscript (not an abstract or dissertation), (4) assessed ART adherence or nonadherence and/or compliance, (5) included a measure of alcohol use, (6) included any Black MSM, (7) study setting in the U.S., (8) results presented for Black MSM in the U.S. distinct from other populations, and (9) participants included were 18 years of age or older. After retrieving full-text quantitative and qualitative articles (n = 114), the first author (SC) and a co-author (NP) independently reviewed each article for eligibility based on the inclusion criteria and discussed until consensus was achieved. A total of five papers met inclusion criteria for this review.

## Results

### Characteristics of Included Studies

Of the 114 studies screened for inclusion, five studies met inclusion criteria for inclusion in this review. The majority of screened studies assessed outcomes other than those of interest in this study (n = 61 [i.e., pre-exposure prophylaxis (PrEP), substance use, not including alcohol use)]. Some excluded studies (n = 5) identified Black MSM in their samples but did not report separate outcomes for them. Five studies (dissertations and meeting abstracts) were excluded due to lack of peer review.

### Study Summaries

Detailed information about each study is as follows (Table 1 includes study summaries). Hightow-Weidman et al. [49] analyzed the baseline cross-sectional data collected as part of a randomized controlled trial (RCT) to explore potential sociodemographic, psychosocial, and HIV care-related predictors of outcomes along the HIV care continuum among young Black MSM (YBMSM) in North Carolina. A total of 193 participants who completed baseline data and identified as HIV positive were included in the analysis. In addition to sociodemographic variables, the study assessed psychosocial outcomes such as substance use, including any alcohol use in the past 3 months. The study also assessed relevant HIV care-related outcomes, including ART medication adherence. Medication adherence was measured using a visual analogue scale in which participants selected a value ranging from 0 to 100% to self-report their adherence daily. A dichotomous variable was created such that those reporting ≥ 90% were classified as adherent.

**Table 1:** Characteristics of (n = 5) included studies assessing the association between alcohol use and ART adherence among Black MSM in the U.S

First author, year	Location (Region)	Data collection method	Participants N total sample (N Black)	Measure of alcohol use	Measure of ART adherence	Main Findings: Association between alcohol use and ART adherence
Hightow-Weidman, 2017	North Carolina (South)	Survey	193	Past 3 months	Visual Analogue Scale (VAS): participants select a value ranging from 0–100% to indicate ART adherence Adherence cut point: ≥ 90% classified as adherent	79.3% reported alcohol use 70.8% reported ART adherence No significant association between alcohol use and adherence (Unadjusted POR [0.48, (0.20, 1.17); p = 0.11]
Dworkin, 2020	Chicago, Illinois (Midwest)	Electronic adherence monitoring (Wisepill) Interviews	40	Number of days per week alcohol is consumed	Number of ART doses missed in the past 4 days	60% reported drinking alcohol at least one day or more per week 53% reported ART adherence When ART adherence was defined as < 90, participants self-reported ART adherence ranged from 79 to 82% In the interviews, 1/32 participants reported missing a dose because they went out drinking
Voisin 2017	Chicago, Illinois (Midwest)	Survey	92	Daily or weekly alcohol use in past 3 months	Participants were asked to self-report what percent, from 0 to 100, did they took their medication as prescribed in the last 30 days Adherence cut point: ≥ 90% classified as adherent	67.4% reported daily or weekly alcohol use in the past 3 months 61.4–67.1% reported ART adherence in the past 30 days (baseline) Daily/weekly alcohol consumption in the past three months was not a predictor of high medication adherence among participants in Chicago OR [.150, (.037–.606); p < .01)
Mutchler, 2019	Los Angeles County, California (West)	Survey	209	Problem alcohol use measured via the Rapid Alcohol Screen Test	Participants were asked to estimate the percentage (range 0 to 100) of prescribed HIV medication that they took in the last month Adherence cut point: ≥ 85% classified as adherent	34.2% of all participants reported problem alcohol use; however, 36% of younger Black MSM (under 50) reporting problem alcohol use compared to 30% of older Black MSM (50 or older) 83.32% reported ART adherence [80.67% in younger sample (under 50); 87.87% in older sample (50 or older)] Problem alcohol use was a significant predictor of lower self-reported adherence among the younger sample bivariate: -16.71(4.26) (p < .001); multivariate: − 15.20 (6.40) (p < .05)
Jemmott, 2019	Philadelphia, Pennsylvania (North)	Interviews	27		Participants discussed positive and negative behavioral, normative, and control beliefs associated with taking ART as prescribed	No percentage of alcohol use reported 75% reported ART adherence Negative behavioral beliefs surrounding the interference of alcohol consumption and medication adherence was listed as a factor in the individual’s decision to take, or not take ART as prescribed

Dworkin et al. [50] conducted a mixed methods pilot study (baseline survey and qualitative interviews) to explore acceptability and feasibility of electronic adherence monitoring (EAM) and to inform intervention development in a sample of 40 African American MSM in Chicago, Illinois. The study assessed daily drinking per week (ranging from 0, 1–2, 3–4, 5 or more) and ART adherence using the Wisepill EAM device. Participants were asked to self-report the number of doses of ART missed in the past 4 days (ranging from 0, 1, 2, ≥ 3). Additionally, the qualitative interviews involved calling or contacting participants each time they missed a dose (as recorded by Wisepill) to find out if they really did miss a dose or whether the Wisepill did not record it correctly. At that call, participants were asked (qualitatively) why they missed the dose.

Voisin et al. [51] conducted a latent class analysis examining longitudinal predictors of ART adherence among a sample of 92 young Black MSM in Chicago, Illinois who previously participated in Project nGage. The study assessed daily/weekly alcohol use (baseline) in the last three months and medication adherence via self-report. Participants were asked to report the percent, from 0 to 100 (0 = none of the time; 50 = half of the time; 100 = all of the time) they took their medication as prescribed in the last 30 days.

Mutchler et al. [52] combined baseline data from two studies (Project Mednet and Project Rise) to compare predictors of ART adherence in 209 younger (under 50) and older (50 or older) Black MSM living with HIV in Los Angeles County, California. The study assessed problem alcohol use using the Rapid Alcohol Screen Test and self-report ART medication adherence by asking participants to estimate the percentage of prescribed ART medication that they had taken in the last month.

Jemmott et al. [53] conducted a theory-based (Theory of Planned Behavior) qualitative study to identify modifiable factors associated with HIV Care Continuum outcomes in 27 African American MSM in Philadelphia, Pennsylvania. The study identified behavioral, normative, and control beliefs regarding medication adherence.

### Study Outcomes

Overall, quantitative study outcomes focused on any alcohol use (yes/no), frequency of alcohol use (days/month), and percentage of medication adherence. O outcomes of interest for the qualitative study were reported barriers to ART adherence.

### Alcohol Use Among Black MSM

Two of the studies assessed alcohol use in the past 3 months [49, 51]. One study assessed the number of days alcohol was drunk in the past week [50], while another study evaluated problem alcohol consumption using the Rapid Alcohol Screen Test [52]. The qualitative study did not explicitly measure alcohol use; rather participants discussed various types of drinking during their interviews [53].

Among the three studies that reported frequency of alcohol use as an exposure, 60–70% of participants reported using alcohol daily or weekly in the past three months [49–51]. Mutchler et al. [52] found that 34% of all participants reported problem alcohol use, with 36% of younger Black MSM (under 50) reporting problem alcohol use compared to 30% of older Black MSM (50 or older).

### ART Adherence Among Black MSM

One study assessed medication adherence using a Visual Analogue Scale (VAS) [49], whereas another study monitored adherence using the Wisepill EAM [50]. Medication adherence was assessed via self-report including the number of missed doses in the past 4 days [50] or the percentage of doses (ranging from 0 = took no doses, 100 = never missed a dose) missed in the past 30 days [51, 52]. One study asked participants to report positive and negative behavioral beliefs about taking ART as prescribed [53].

The majority of the studies used different cut-off points for adherence, ranging from 80 to ≥ 90% [49–52]. When ART adherence was defined as < 90, Black MSM reported ART adherence ranging from 79 to 82% [50]. One study examined whether adherence was different in younger (under 50) vs. older (50 or older) Black MSM [52]. The authors found that 83% of the total sample reported ≥ 85% adherence, with 80% of young Black MSM reporting ≥ 85% adherence compared to 87% of older Black MSM [52].

Using the VAS, one study found approximately 70% of participants reported ≥ 90% medication adherence [49]. Another longitudinal study reported that 53% of participants missed doses of ART in the past four days at baseline [50]. An estimated 61–67% of participants in the Voisin et al. [51] study reported taking their medication 90% of the time in the past 30 days. Another study found that 75% of participants reported taking ART as prescribed [53].

### Association Between Alcohol Use and ART Adherence Among Black MSM

In bivariate analyses, two studies reported significant associations between alcohol use and ART adherence. One study reported that problem alcohol use (p < 0.001) was associated with lower self-reported ART adherence among Black MSM in Los Angeles [52]. When examining alcohol use and ART adherence among younger vs. older Black MSM, the authors found that alcohol use remained a significant predictor of lower self-reported ART adherence in younger vs. older Black MSM in both the bivariate − 16.71 (4.26) (p < 0.001) and multivariate models − 15.20 (6.40) (p < 0.05) [52]. Using latent class analyses, Voisin et al. [51] found that daily/weekly alcohol consumption in the past 3 months was not a predictor of high medication adherence among participants in Chicago (OR 0.150, [0.037–0.606)] p < 0.01). Likewise, in bivariate and multivariate analyses of self-report ART adherence (≥ 90), past-3-month alcohol use was not associated with a lower risk of self-reported ART adherence (≥ 90) among Black MSM in North Carolina (Unadjusted POR [0.48, (0.20, 1.17); p = 0.11] [49].

Two studies did not quantitatively assess the association between alcohol use and ART adherence. Dworkin et al. [50] found that only 1/32 participants in Chicago reported missing a dose of ART because they went out drinking. The qualitative study conducted in Philadelphia by Jemmott et al. [53] found that negative behavioral beliefs surrounding the use of ART during alcohol use and resulting side effects were listed as a factor in the individual’s decision to take or not take ART as prescribed.

## Discussion

Our review systematically reviewed and summarized the evidence regarding the association between alcohol use and ART adherence among Black MSM in U.S., with particular attention on the southern U.S. The relative dearth of research on this topic is particularly unfortunate given evidence of HIV-related disparities in the U.S. and in the South, Moreover, three of the five included studies identified alcohol as a barrier to ART adherence among a sample of Black MSM in the U.S. but not in the South [51, 52]. We believe this to be the only systematic literature review that focused solely on alcohol use and medication adherence among HIV-positive Black MSM in the U.S.

### Gaps in Literature

Our review has noted several gaps in the literature that highlight possible directions for future research. One of these is the need for more experimental study designs that examine the association among alcohol use and ART adherence (including racial/ethnic differences) and treatment effectiveness among Black MSM in U.S. and specifically in the South. Another is the need to separate data for subpopulations such as Black MSM in multi-site studies. While many multi-site studies included Southern cities as sites, they did not separate results by city. Additionally, most of the excluded studies were observational and the majority of the included studies were cross-sectional [49, 51, 52], qualitative [53], or mixed methods [50].

Regional inequalities in state and federal funding as well as NIH funding for HIV prevention and care, specifically in the Deep South have been documented [14]. For example, PLWH in the Deep South have been shown to receive less federal funding per person compared to PLWH in other U.S. regions/areas [14]. To combat the disparate rates of HIV infection in the South, the CDC and Health Resources and Services Administration (HRSA) have made recent changes to their budgets for resources and programs [10, 14, 16]. In 2017, the CDC hosted its first “HIV in the South” town hall meeting to gain insight into the perspectives and opinions of key stakeholders and thus inform its HIV prevention efforts in the South [10, 16]. Participants discussed barriers and facilitators to addressing HIV prevention in the South yet seem to have made little to no mention of substance misuse in general among PLWH, and nothing specifically about alcohol use among Black MSM [10, 16]. Differences in funding could very plausibly explain the lack of research focused on alcohol-using Black MSM in the South, as well as in HIV disparities in the South.

### Recommendations for Future Research

Two of the goals of our review were to summarize the existing evidence and to provide recommendations for using alcohol-related behavioral research to build stronger HIV preventive interventions for this population. Previous research examining interventions that address substance use and sexual risk behaviors have reported mixed findings or limited success [54–56]. Our review failed to discover any publications identifying alcohol-related interventions focused on improving ART adherence among Black MSM in the U.S. or in the South. To increase the effectiveness of evidence-based interventions and best practices for HIV prevention and develop new interventions, some researchers have called for interventions that address multiple levels of barriers: individual, societal, and policy [57].

In addition, alcohol-related behavioral research could prove an important new direction in the study of HIV risk behaviors in Black MSM and in advancing HIV prevention interventions that target this population in at least two ways. First, alcohol-related behavioral research could empirically establish causal connections between alcohol consumption and HIV-related risk behaviors in Black MSM, including the mechanisms that underlie them. Jemmott et al. [53] provided some insight into a potential mechanism associated with alcohol use and ART adherence, but this could be expanded. While the participants did not mention “forgetting” or “passing out drunk” due to alcohol consumption as a barrier to adherence, they did mention that alcohol and ART can lead to side effects so they actively avoid ART on days when they are going to “party”. Such knowledge identifies essential prevention intervention targets [58]. As mentioned, Black MSM in the South continue to have the least favorable HIV care continuum (HCC) outcomes including ART initiation, retention in care, and achieving viral suppression [15, 59] but none of CDC’s four tailored interventions specifically includes an alcohol component [42–45]. Determining the degree and contexts in which alcohol is influential at each phase of the HIV care continuum [56] among Black MSM in the South is important. The authors believe this can be accomplished by applying principles and methods of big data to better understand the effects of specific contextual factors experienced by Black MSM in the South along the HIV care continuum.

Second, alcohol-related behavioral research can help to advance knowledge about the individual differences and contextual variables that might alter alcohol’s association to HIV-related risk behaviors [38, 55]. It may also help to develop more person-centered HIV prevention interventions specifically designed for Black MSM in the South who consume alcohol. Expanding alcohol-related behavioral research beyond individual-level factors may help us to understand how alcohol might affect individual HIV-related decision-making within specific contexts as well as strengthen our understanding of specific contextual variables that may be more distal as well as proximal to the decision-making event [38]. This could require a program of research that integrates observational studies with experimental studies such that the design of each is informed by theory and empirical findings from the other. Pursuing this research further could possibly involve applying principles and methods of “just-in-time” interventions and machine learning to the ARBR sexual risk agenda [60] since just-in-time interventions provide real-time support when needed or when the participant is most receptive (as demonstrated by completion of ongoing assessments of the participant’s unique risk factors, internal state, and context) [60–62]. Since most just-in-time interventions rely on smartphones [63], researchers could potentially collect data on a wide range on sexual risk behaviors in the natural environment that could generate ideas about mechanisms specific to the South and then could be tested by use of observational or experimental studies.

## Limitations

We note several potential limitations as context for interpreting our findings. First, our study selection sought papers that focused exclusively on Black MSM in the South and later revised the inclusion criteria to include Black MSM in the U.S. In addition, some papers did not clearly distinguish between exclusive alcohol use and alcohol use in combination with use of other substances. The impact of other drug use may be similar to the effects of alcohol on HIV risk in Southern Black MSM, but we cannot tell from these data. Our focus on Black MSM in the South may have limited generalizability to non-Black MSM or Black MSM who do not live in the U.S. However, we believe that many of the findings and suggestions should be relevant to those other populations and settings.

Due to the limited amount of research examining the association between alcohol and ART adherence, we are unable to determine alcohol’s role as either a mediator or moderator of ART adherence among Black MSM in the U.S. or in the South. The literature has yielded evidence of direct causal mechanisms by which alcohol use is associated with ART adherence in this population. Unlike previously conducted systematic reviews examining HIV-related disparities in Black MSM or assessing the role of alcohol and ART adherence in PWH, our review focused on the intersection of race and alcohol use in the context of HIV infection: namely, Black MSM with documented HIV and reported drinking. Given that Black MSM in the U.S.—specifically the South—are disproportionately affected by HIV, remain at high risk for HIV transmission, and are less likely to adhere to ART, this review exposes a major gap in the literature upon which to build effective interventions and formulate solid policy.

## Conclusions

Black MSM in the South are experiencing the highest rates of new HIV infections in the U.S. Whereas previous reviews on factors responsible for this disparity have not isolated alcohol use, the sole study that met the regional criterion for inclusion in our review did not find an association between alcohol use and ART adherence among a sample of Black MSM. Interventions to address the association between alcohol use and ART adherence could include approaches that focus on individual change (e.g., reduction in drinking) as well as assessing contextual factors that could also influence drinking behavior, such as social networks and high-risk partners, settings where drinking and HIV transmission risk overlap, community services and policies that are welcoming to Black MSM in the South and that can address both alcohol use and HIV-related health concerns.

## Supplementary Information

Below is the link to the electronic supplementary material.

Supplementary file1 (DOCX 16 KB)

## References

[1] Millett GA, Peterson JL, Wolitski RJ, Stall R (2006). Greater risk for HIV infection of black men who have sex with men: a critical literature review. Am J Public Health.

[2] Millett GA, Flores SA, Peterson JL, Bakeman R (2007). Explaining disparities in HIV infection among black and white men who have sex with men: a meta-analysis of HIV risk behaviors. AIDS.

[3] Maulsby C, Millett G, Lindsey K (2014). HIV among black men who have sex with men (MSM) in the United States: a review of the literature. AIDS Behav.

[4] Woolf-King SE, Sheinfil AZ, Ramos J (2020). A conceptual model of alcohol use and adherence to antiretroviral therapy: systematic review and theoretical implications for mechanisms of action. Health Psychol Rev.

[5] Tobin K, Davey-Rothwell M, Yang C, Siconolfi D, Latkin C (2014). An examination of associations between social norms and risky alcohol use among African American men who have sex with men. Drug Alcohol Depend.

[6] Tobin KE, Yang C, King K, Latkin CA, Curriero FC (2016). Associations between drug and alcohol use patterns and sexual risk in a sample of African American men who have sex with men. AIDS Behav.

[7] Wray TB, Pantalone DW, Kahler CW, Monti PM, Mayer KH (2016). The role of discrimination in alcohol-related problems in samples of heavy drinking HIV-negative and positive men who have sex with men (MSM). Drug and Alcohol Depend.

[8] Washington TA, Patel SN, Meyer-Adams N (2017). Drinking patterns and HIV risk behaviors among Black and Latino men who have sex within Los Angeles County. Am J Mens Health.

[9] Matthews DD, Herrick AL, Coulter RW (2016). Running backwards: consequences of current HIV incidence rates for the next generation of black MSM in the United States. AIDS Behav.

[10] McCree DH, Williams AM, Chesson HW (2019). Changes in disparities in estimated HIV incidence rates among black, Hispanic/Latino, and white men who have sex with men (MSM) in the United States, 2010–2015. J Acquir Immune Defic Syndr.

[11] Prejean J, Tang T, Hall HI (2013). HIV diagnoses and prevalence in the southern region of the United States, 2007–2010. J Community Health.

[12] Reif S, Pence BW, Hall I, Hu X, Whetten K, Wilson E (2015). HIV diagnoses, prevalence and outcomes in nine southern states. J Community Health.

[13] Gray SC, Massaro T, Chen I (2016). A county-level analysis of persons living with HIV in the southern United States. AIDS Care.

[14] Reif S, Safley D, McAllaster C, Wilson E, Whetten K (2017). State of HIV in the US deep south. J Community Health.

[15] Carter JW, Flores SA (2019). Improving the HIV prevention landscape to reduce disparities for Black MSM in the South. AIDS Behav.

[16] Jeffries WL, Henny KD (2019). From epidemiology to action: the case for addressing social determinants of health to end HIV in the Southern United States. AIDS Behav.

[17] Watson M, Johnson SD, Zhang T, Oster AM (2019). Characteristics of and trends in HIV diagnoses in the Deep South region of the United States, 2012–2017. AIDS Behav.

[18] Colasanti JA, Armstrong WS (2019). Challenges of reaching 90–90–90 in the Southern United States. Curr Opin HIV AIDS.

[19] Oster AM, Wiegand RE, Sionean C (2011). Understanding disparities in HIV infection between black and white MSM in the United States. AIDS.

[20] Millett GA, Peterson JL, Flores SA (2012). Comparisons of disparities and risks of HIV infection in black and other men who have sex with men in Canada, UK, and USA: a meta-analysis. Lancet.

[21] Algarin AB, Zhou Z, Cook CL, Cook RL, Ibañez GE (2019). Age, sex, race, ethnicity, sexual orientation: intersectionality of marginalized-group identities and enacted HIV-related stigma among people living with HIV in Florida. AIDS Behav.

[22] Maulsby C, Millett G, Lindsey K (2013). A systematic review of HIV interventions for black men who have sex with men (MSM). BMC Public Health.

[23] Centers for Disease Control and Prevention. Understanding the HIV care continuum, 2019 [Internet]. Atlanta (GA): Centers for Disease Control and Prevention. 2019. https://www.cdc.gov/hiv/pdf/library/factsheets/cdc-hiv-care-continuum.pdf. Accessed on 26 Apr 2021

[24] Hogg RS (2018). Understanding the HIV care continuum. Lancet HIV.

[25] Hendershot CS, Stoner SA, Pantalone DW, Simoni JM (2009). Alcohol use and antiretroviral adherence: review and meta-analysis. J Acquir Immune Defic Syndr.

[26] Bryant KJ, Nelson S, Braithwaite RS, Roach D (2010). Integrating HIV/AIDS and alcohol research. Alcohol Res Health.

[27] Grodensky CA, Golin CE, Ochtera RD, Turner BJ (2012). Systematic review: effect of alcohol intake on adherence to outpatient medication regimens for chronic diseases. J Stud Alcohol Drugs.

[28] Vagenas P, Azar MM, Copenhaver MM, Springer SA, Molina PE, Altice FL (2015). The impact of alcohol use and related disorders on the HIV continuum of care: a systematic review. Curr HIV/AIDS Rep.

[29] Ge S, Sanchez M, Nolan M, Liu T, Savage CL (2018). Is alcohol use associated with increased risk of developing adverse health outcomes among adults living with human immunodeficiency virus: a systematic review. J Addict Nurs.

[30] Costa JD, Torres TS, Coelho LE, Luz PM (2018). Adherence to antiretroviral therapy for HIV/AIDS in Latin America and the Caribbean: systematic review and meta-analysis. J Int AIDS Soc.

[31] Velloza J, Kemp CG, Aunon FM, Ramaiya MK, Creegan E, Simoni JM (2020). Alcohol use and antiretroviral therapy non-adherence among adults living with HIV/AIDS in Sub-Saharan Africa: a systematic review and meta-analysis. AIDS Behav.

[32] Centers for Disease Control and Prevention. HIV treatment as prevention [Internet]. Atlanta (GA): Centers for Disease Control and Prevention. 2021. https://www.cdc.gov/hiv/risk/art/index.html. Accessed on 26 Apr 2021

[33] Hahn JA, Samet JH (2010). Alcohol and HIV disease progression: weighing the evidence. Curr HIV/AIDS Rep.

[34] Deiss RG, Mesner O, Agan BK (2016). Characterizing the association between alcohol and HIV virologic failure in a military cohort on antiretroviral therapy. Alcohol Clin Exp Res.

[35] Cook RL, Zhou Z, Miguez MJ (2019). Reduction in drinking was associated with improved clinical outcomes in women with HIV infection and unhealthy alcohol use: results from a randomized clinical trial of oral naltrexone versus placebo. Alcohol Clin Exp Res.

[36] Woolf SE, Maisto SA (2009). Alcohol use and risk of HIV infection among men who have sex with men. AIDS Behav.

[37] Rosen MI, Black AC, Arnsten JH (2013). Association between use of specific drugs and antiretroviral adherence: findings from MACH 14. AIDS Behav.

[38] Maisto SA, Simons JS (2016). Research on the effects of alcohol and sexual arousal on sexual risk in men who have sex with men: implications for HIV prevention interventions. AIDS Behav.

[39] Zhang C, Qian HZ, Yin L (2016). Sexual behaviors linked to drug and alcohol use among men who have sex with men in China. Subst Use Misuse.

[40] Rehm J, Probst C, Shield KD, Shuper PA (2017). Does alcohol use have a causal effect on HIV incidence and disease progression? A review of the literature and a modeling strategy for quantifying the effect. Population Health Metr.

[41] Centers for Disease Control and Prevention. Compendium of evidence-based interventions and best practices for HIV prevention [Internet]. Atlanta (GA): Centers for Disease Control and Prevention. 2021. https://www.cdc.gov/hiv/research/interventionresearch/compendium/index.html. Accessed on 29 Apr 2021

[42] Wilton L, Herbst JH, Coury-Doniger P (2009). Efficacy of an HIV/STI prevention intervention for black men who have sex with men: findings from the Many Men, Many Voices (3MV) project. AIDS Behav.

[43] Hightow-Weidman LB, Smith JC, Valera E, Matthews DD, Lyons P (2011). Keeping them in “STYLE”: finding, linking, and retaining young HIV-positive black and Latino men who have sex with men in care. AIDS Patient Care STDs.

[44] Bouris A, Jaffe K, Eavou R (2017). Project nGage: results of a randomized controlled trial of a dyadic network support intervention to retain young Black men who have sex with men in HIV care. AIDS Behav.

[45] Desrosiers A, Levy M, Dright A (2019). A randomized controlled pilot study of a culturally-tailored counseling intervention to increase uptake of HIV pre-exposure prophylaxis among young Black men who have sex with men in Washington. DC AIDS Behav.

[46] Moher D, Liberati AA, Tetzlaff J, Altman DG (2009). Preferred reporting items for systematic reviews and meta-analyses: the PRISMA statement. BMJ.

[47] Page MJ, McKenzie JE, Bossuyt PM, Boutron I, Hoffmann TC, Mulrow CD (2021). The PRISMA 2020 statement: an updated guideline for reporting systematic reviews. BMJ.

[48] Covidence systematic review software, 2021 [Internet], Melbourne (Australia): Veritas Health Innovation. 2021. www.covidence.org. Accessed on 11 Apr 2021

[49] Hightow-Weidman L, LeGrand S, Choi SK, Egger J, Hurt CB, Muessig KE (2017). Exploring the HIV continuum of care among young black MSM. PLoS ONE.

[50] Dworkin MS, Panchal P, Wiebel W, Garofalo R, Jimenez A, Haberer JE (2020). Experience with antiretroviral electronic adherence monitoring among young African American men who have sex with men living with HIV: findings to inform a triaged real-time alert intervention. AIDS Care.

[51] Voisin DR, Quinn K, Kim DH, Schneider J (2017). A longitudinal analysis of antiretroviral adherence among young Black men who have sex with men. J Adolesc Health.

[52] Mutchler MG, Bogart LM, Klein DJ (2019). Age matters: differences in correlates of self-reported HIV antiretroviral treatment adherence between older and younger Black men who have sex with men living with HIV. AIDS Care.

[53] Jemmott JB, Zhang J, Croom M, Icard LD, Rutledge SE, O’Leary A (2019). Barriers and facilitators to engaging African American men who have sex with men in the HIV care continuum: a theory-based qualitative study. J Assoc Nurses AIDS Care.

[54] Jackson C, Geddes R, Haw S, Frank J (2012). Interventions to prevent substance use and risky sexual behaviour in young people: a systematic review. Addiction.

[55] Bourne A, Weatherburn P (2017). Substance use among men who have sex with men: patterns, motivations, impacts and intervention development need. Sexually Transm Infect.

[56] Williams EC, Hahn JA, Saitz R, Bryant K, Lira MC, Samet JH (2016). Alcohol use and human immunodeficiency virus (HIV) infection: current knowledge, implications, and future directions. Alcohol Clin Exp Res.

[57] Carrasco MA, Esser MB, Sparks A, Kaufman MR (2016). HIV-alcohol risk reduction interventions in Sub-Saharan Africa: a systematic review of the literature and recommendations for a way forward. AIDS Behav.

[58] Kazdin AE (2008). Evidence-based treatment and practice: new opportunities to bridge clinical research and practice, enhance the knowledge base, and improve patient care. Am Psychol.

[59] Centers for Disease Control and Prevention. Division of HIV/AIDS prevention HIV in the South meeting notes [Internet]. Atlanta (GA): Centers for Disease Control and Prevention. 2017. https://www.cdc.gov/hiv/pdf/dhap/cdc-hiv-in-the-south-meeting.pdf. Accessed on 20 Apr 2021

[60] Nahum-Shani I, Smith SN, Spring BJ (2018). Just-in-time adaptive interventions (JITAIs) in mobile health: key components and design principles for ongoing health behavior support. Ann Behav Med.

[61] Mustanski B (2007). The influence of state and trait affect on HIV risk behaviors: a daily diary study of MSM. Health Psychol.

[62] Wray TB, Kahler CW, Monti PM (2016). Using ecological momentary assessment (EMA) to study sex events among very high-risk men who have sex with men (MSM). AIDS Behav.

[63] Goldstein SP, Evans BC, Flack D (2017). Return of the JITAI: applying a just-in-time adaptive intervention framework to the development of m-health solutions for addictive behaviors. Int J Behav Med.

