# Supplementary material for: The Relationship of Alcohol to ART Adherence Among Black MSM in the U.S.: Is it Any Different Among Black MSM in the South?
Source: AIDS Behav. 2021 Nov 6;25(Suppl 3):302–13. doi: 10.1007/s10461-021-03479-3 (PMC8610946; doi:10.1007/s10461-021-03479-3)
Supplement: Supplementary file 1 — Supplementary file1 (DOCX 16 KB) [file 10461_2021_3479_MOESM1_ESM.docx]

**Supplement I. Search Strategy for Each Database**

| **Data name (Provider/Platform)** | **Search strategy** | **Limits** |
| --- | --- | --- |
| **Academic Search Premier (Ebsco)** | (((DE "AFRICAN Americans" OR DE "BLACK people” OR (TI "african american*" OR afroamerican* OR "black men" OR "black man" OR "black population" OR "black people" OR "black person*" OR "black race" OR "racially black" OR "black homosexual*" OR "black transsexual*" OR "black trans-sexual*" OR "black trans sexual*"OR "black bisexual*" OR "black bi-sexual*" OR "black bi sexual*" OR "black gay*" OR "gay black*" OR AB "african american*" OR afroamerican* OR "black men" OR "black man" OR "black population" OR "black people" OR "black person*" OR "black race" OR "racially black" OR "black homosexual*" OR "black transsexual*" OR "black trans-sexual*" OR "black trans sexual*"OR "black bisexual*" OR "black bi-sexual*" OR "black bi sexual*" OR "black gay*" OR "gay black*")) AND (DE "GAY men" OR DE "BEARS (Gay culture)" OR DE "SEXUAL minority men" OR DE "TRANS men" OR DE "HEALTH of gay men" OR DE "MALE homosexuality" OR (DE "BLACK LGBTQ people" AND DE "Men") OR (TI "men who have sex with men" OR "MSM" OR "male homosexual*" OR "homosexual male*" OR AB "men who have sex with men" OR "MSM" OR "male homosexual*" OR "homosexual male*" OR TI ((men OR man OR male*) AND (gay OR gays OR bisexual* OR "bi-sexual*" OR "bi sexual*" OR queer OR queers OR "trans-sexual*" OR "trans sexual*" OR transsexual*)) OR AB ((men OR man OR male*) AND (gay OR gays OR bisexual* OR "bi-sexual*" OR "bi sexual*" OR queer OR queers OR "trans-sexual*" OR "trans sexual*" OR transsexual*))) AND (DE "DRUGS" OR DE "ALCOHOL" OR DE "CONTROLLED drugs" OR DE "CORTICOSTEROIDS" OR DE "NEUROPROTECTIVE agents" OR DE "NONPRESCRIPTION drugs" OR DE "ORAL medication" OR DE "PSYCHIATRIC drugs" OR DE "RESPIRATORY agents" OR DE "SYNTHETIC drugs") AND (DE "PATIENT compliance" OR DE "PATIENT decision making" OR DE "SELF-efficacy")  AND (TI "medication adheren*" OR "medication adheran*" OR "medication complian*" OR "medicine adheren*" OR "medicine adheran*" OR "medicine complian*" OR "drug adheren*" OR "drug adheran*" OR "drug complian*" OR "medication nonadheren*" OR "medication nonadheran*" OR "medication noncomplian*" OR "medication non-adheren*" OR "medication non-adheran*" OR "medication non-complian*" OR "medicine nonadheren*" OR "medicine nonadheran*" OR "medicine non-adheren*" OR "medicine non-adheran*" OR "medicine noncomplian*" OR "medicine non-complian*" OR "drug noncomplian*" OR "drug non-complian*" OR "drug nonadheren*" OR "drug nonadheran*" OR "drug non-adheren*" OR "drug non-adheran*" OR "drug noncomplian*" OR "drug non-complian*" OR (("patient adheren*" OR "patient adheran*" OR "patient nonadheren*" OR "patient nonadheran*" OR "patient non-adheren*" OR "patient non-adheran*" OR "patient complian*" OR "patient noncomplian*" OR "patient non-complian*") AND (pharmac* OR drug* OR medication* OR medicine* OR "ART" OR "antiretroviral therap*" OR "anti-retroviral therap*" OR PReP OR "pre-exposure prophylaxis" OR "pre exposure prophylaxis" OR "preexposure prophylaxis")) OR AB "medication adheren*" OR "medication adheran*" OR "medication complian*" OR "medicine adheren*" OR "medicine adheran*" OR "medicine complian*" OR "drug adheren*" OR "drug adheran*" OR "drug complian*" OR "medication nonadheren*" OR "medication nonadheran*" OR "medication noncomplian*" OR "medication non-adheren*" OR "medication non-adheran*" OR "medication non-complian*" OR "medicine nonadheren*" OR "medicine nonadheran*" OR "medicine non-adheren*" OR "medicine non-adheran*" OR "medicine noncomplian*" OR "medicine non-complian*" OR "drug noncomplian*" OR "drug non-complian*" OR "drug nonadheren*" OR "drug nonadheran*" OR "drug non-adheren*" OR "drug non-adheran*" OR "drug noncomplian*" OR "drug non-complian*" OR (("patient adheren*" OR "patient adheran*" OR "patient nonadheren*" OR "patient nonadheran*" OR "patient non-adheren*" OR "patient non-adheran*"OR "patient complian*" OR "patient noncomplian*" OR "patient non-complian*") AND (pharmac* OR drug* OR medication* OR medicine* OR "ART" OR "antiretroviral therap*" OR "anti-retroviral therap*" OR PReP OR "pre-exposure prophylaxis" OR "pre exposure prophylaxis" OR "preexposure prophylaxis"))) AND (DE "HIV" OR DE "MULTIDRUG-resistant HIV" OR DE "AIDS" OR DE "HIGHLY active antiretroviral therapy" OR DE "HIV antibodies" OR DE "HIV infections" OR DE "AIDS" OR DE "AIDS & insurance" OR DE "AIDS education" OR DE "AIDS immunology" OR DE "AIDS patients" OR DE "AIDS prevention" OR DE "AIDS vaccines" OR DE "AIDS-related complex" OR (TI "human immunodeficiency virus" OR "HIV" OR "Acquired Immunodeficiency Syndrome" OR ("AIDS" NOT ("communication aids" OR "visual aids" OR "teaching aids" OR "mobility aids" OR "incontinence aids" OR "ambulation aids" OR "hearing aids" OR sensory aids"))) OR AB "human immunodeficiency virus" OR "HIV" OR "Acquired Immunodeficiency Syndrome" OR ("AIDS" NOT ("communication aids" OR "visual aids" OR "teaching aids" OR "mobility aids" OR "incontinence aids" OR "ambulation aids" OR "hearing aids" OR sensory aids"))) | Published Date: 20100101-20211231; Language: English |
| **APA PsycINFO (Ebsco)** | (DE "Human Males" AND ( DE "Male Homosexuality" OR DE "LGBTQ" OR DE "Same Sex Couples" OR DE "Same Sex Marriage" OR DE "Sexual Minority Groups" OR DE "Transsexualism")) OR (TI ("men who have sex with men" OR "MSM" OR "male homosexual*" OR "homosexual male*") OR AB ("men who have sex with men" OR "MSM" OR "male homosexual*" OR "homosexual male*" )) OR (TI ((men OR man OR male*) AND (gay OR gays OR bisexual* OR "bi-sexual*" OR "bi sexual*" OR queer OR queers OR "trans-sexual*" OR "trans sexual*" OR transsexual*)) OR AB ((men OR man OR male*) AND (gay OR gays OR bisexual* OR "bi-sexual*" OR "bi sexual*" OR queer OR queers OR "trans-sexual*" OR "trans sexual*" OR transsexual*)) ) ) AND (DE "African Cultural Groups" OR DE "Blacks" OR TI ( "african american*" OR afroamerican* OR "black men" OR "black man" OR "black population" OR "black people" OR "black person*" OR "black race" OR "racially black" OR "black homosexual*" OR "black transsexual*" OR "black trans-sexual*" OR "black trans sexual*"OR "black bisexual*" OR "black bi-sexual*" OR "black bi sexual*" OR "black gay*" OR "gay black*" ) OR AB ( "african american*" OR afroamerican* OR "black men" OR "black man" OR "black population" OR "black people" OR "black person*" OR "black race" OR "racially black" OR "black homosexual*" OR "black transsexual*" OR "black trans-sexual*" OR "black trans sexual*"OR "black bisexual*" OR "black bi-sexual*" OR "black bi sexual*" OR "black gay*" OR "gay black*" )) OR (DE "HIV" OR DE "AIDS" OR DE "AIDS Dementia Complex" OR DE "AIDS Prevention" OR DE "Pre-Exposure Prophylaxis" OR TI "human immunodeficiency virus" OR "HIV" OR "Acquired Immunodeficiency Syndrome" OR ("AIDS" NOT ("communication aids" OR "visual aids" OR "teaching aids") OR AB "human immunodeficiency virus" OR "HIV" OR "Acquired Immunodeficiency Syndrome" OR ("AIDS" NOT ("communication aids" OR "visual aids" OR "teaching aids"))) AND (DE "Treatment Compliance" OR DE "Medication-Assisted Treatment" OR DE "Methadone Maintenance" OR DE "Treatment Dropouts" OR DE "Treatment Refusal" OR DE "Maintenance Therapy" OR DE "Treatment Termination" OR DE "Client Participation" OR DE "Self-Medication" OR (DE "Compliance" AND (DE "Drug Therapy" OR DE "Drug Usage" OR DE "Pre-Exposure Prophylaxis")) OR TI "drug adheren*" OR "drug adheran*" OR "drug complian*" OR "medication nonadheren*" OR "medication nonadheran*" OR "medication noncomplian*" OR "medication non-adheren*" OR "medication non-adheran*" OR "medication non-complian*" OR "medicine nonadheren*" OR "medicine nonadheran*" OR "medicine non-adheren*" OR "medicine non-adheran*" OR "medicine noncomplian*" OR "medicine non-complian*" OR "drug noncomplian*" OR "drug non-complian*" OR "drug nonadheren*" OR "drug nonadheran*" OR "drug non-adheren*" OR "drug non-adheran*" OR "drug noncomplian*" OR "drug non-complian*" OR (("patient adheren*" OR "patient adheran*" OR "patient nonadheren*" OR "patient nonadheran*" OR "patient non-adheren*" OR "patient non-adheran*"OR "patient complian*" OR "patient noncomplian*" OR "patient non-complian*") AND (pharmac* OR drug* OR medication* OR medicine* OR "ART" OR "antiretroviral therap*" OR "anti-retroviral therap*" OR "PReP" OR "pre-exposure prophylaxis" OR "pre exposure prophylaxis" OR "preexposure prophylaxis")) ) OR AB ( "drug adheren*" OR "drug adheran*" OR "drug complian*" OR "medication nonadheren*" OR "medication nonadheran*" OR "medication noncomplian*" OR "medication non-adheren*" OR "medication non-adheran*" OR "medication non-complian*" OR "medicine nonadheren*" OR "medicine nonadheran*" OR "medicine non-adheren*" OR "medicine non-adheran*" OR "medicine noncomplian*" OR "medicine non-complian*" OR "drug noncomplian*" OR "drug non-complian*" OR "drug nonadheren*" OR "drug nonadheran*" OR "drug non-adheren*" OR "drug non-adheran*" OR "drug noncomplian*" OR "drug non-complian*" OR (("patient adheren*" OR "patient adheran*" OR "patient nonadheren*" OR "patient nonadheran*" OR "patient non-adheren*" OR "patient non-adheran*"OR "patient complian*" OR "patient noncomplian*" OR "patient non-complian*") AND (pharmac* OR drug* OR medication* OR medicine* OR "ART" OR "antiretroviral therap*" OR "anti-retroviral therap*" OR "PReP" OR "pre-exposure prophylaxis" OR "pre exposure prophylaxis" OR "preexposure prophylaxis")) ) | Pub Date 2010->, English, Humans, Peer-reviewed, Male, Transgender,  Academic journals;  Exclude Dissertations, Professional |
| **CINAHL (Ebsco)** | **(**MH "Blacks" OR TI ("african american*" OR afroamerican* OR "black men" OR "black man" OR "black population" OR "black people" OR "black person*" OR "black race" OR "racially black" OR "black homosexual*" OR "black transsexual*" OR "black trans-sexual*" OR "black trans sexual*"OR "black bisexual*" OR "black bi-sexual*" OR "black bi sexual*" OR "black gay*" OR "gay black*") OR AB ("african american*" OR afroamerican* OR "black men" OR "black man" OR "black population" OR "black people" OR "black person*" OR "black race" OR "racially black" OR "black homosexual*" OR "black transsexual*" OR "black trans-sexual*" OR "black trans sexual*"OR "black bisexual*" OR "black bi-sexual*" OR "black bi sexual*" OR "black gay*" OR "gay black*")) AND (MH "Human Immunodeficiency Virus+" OR MH "HIV Infections+" OR MH "HIV-Infected Patients+" OR MH "Anti-HIV Agents+" OR MH "Acquired Immunodeficiency Syndrome" OR MH "AIDS Patients" OR MH "AIDS-Related Complex" OR MH "HIV Wasting Syndrome" OR MH "HIV Seropositivity" OR MH "HIV Enteropathy" OR MH "AIDS-Related Opportunistic Infections" OR MH "AIDS Dementia Complex" OR MH "AIDS-Associated Nephropathy" OR MH "AIDS Serodiagnosis" OR MH "HIV Entry and Fusion Inhibitors+") OR (TI "human immunodeficiency virus" OR "HIV" OR "Acquired Immunodeficiency Syndrome" OR ("AIDS" NOT ("communication aids" OR "visual aids" OR "teaching aids" OR "sensory aids" OR "hearing aids" OR "ambulation aids" OR "incontinence aids")) OR AB "human immunodeficiency virus" OR "HIV" OR "Acquired Immunodeficiency Syndrome" OR ("AIDS" NOT ("communication aids" OR "visual aids" OR "teaching aids" OR "sensory aids" OR "hearing aids" OR "ambulation aids" OR "incontinence aids"))) AND (MH "Medication Compliance" OR MH "Compliance with Medication Regimen (Saba CCC)" OR OR MH "Noncompliance of Medication Regimen (Saba CCC)" OR ((MH "Noncompliance of Therapeutic Regimen (Saba CCC)" OR MH "Compliance with Therapeutic Regimen (Saba CCC)" OR MH "Patient Compliance" OR MH "Compliance Behavior (Iowa NOC)" OR OR MH "Compliance with Medical Regimen (Saba CCC)" OR MH "Compliance with Therapeutic Regimen (Saba CCC)" OR MH "Compliance with Safety Precautions (Saba CCC)" OR MH "Compliance Care (Saba CCC)" OR MH "Treatment Refusal") AND (MH "Medication Management" OR MH "Medication Treatment (Saba CCC)" OR MH "Medication Regimen (Omaha)")) OR TI "medication adheren*" OR "medication adheran*" OR "medication complian*" OR "medicine adheren*" OR "medicine adheran*" OR "medicine complian*" OR "drug adheren*" OR "drug adheran*" OR "drug complian*" OR "medication nonadheren*" OR "medication nonadheran*" OR "medication noncomplian*" OR "medication non-adheren*" OR "medication non-adheran*" OR "medication non-complian*" OR "medicine nonadheren*" OR "medicine nonadheran*" OR "medicine non-adheren*" OR "medicine non-adheran*" OR "medicine noncomplian*" OR "medicine non-complian*" OR "drug noncomplian*" OR "drug non-complian*" OR "drug nonadheren*" OR "drug nonadheran*" OR "drug non-adheren*" OR "drug non-adheran*" OR "drug noncomplian*" OR "drug non-complian*" OR (("patient adheren*" OR "patient adheran*" OR "patient nonadheren*" OR "patient nonadheran*" OR "patient non-adheren*" OR "patient non-adheran*"OR "patient complian*" OR "patient noncomplian*" OR "patient non-complian*") AND (pharmac* OR drug* OR medication* OR medicine* OR "ART" OR "antiretroviral therap*" OR "anti-retroviral therap*" OR PReP OR "pre-exposure prophylaxis" OR "pre exposure prophylaxis" OR "preexposure prophylaxis")) OR AB "medication adheren*" OR "medication adheran*" OR "medication complian*" OR "medicine adheren*" OR "medicine adheran*" OR "medicine complian*" OR "drug adheren*" OR "drug adheran*" OR "drug complian*" OR "medication nonadheren*" OR "medication nonadheran*" OR "medication noncomplian*" OR "medication non-adheren*" OR "medication non-adheran*" OR "medication non-complian*" OR "medicine nonadheren*" OR "medicine nonadheran*" OR "medicine non-adheren*" OR "medicine non-adheran*" OR "medicine noncomplian*" OR "medicine non-complian*" OR "drug noncomplian*" OR "drug non-complian*" OR "drug nonadheren*" OR "drug nonadheran*" OR "drug non-adheren*" OR "drug non-adheran*" OR "drug noncomplian*" OR "drug non-complian*" OR (("patient adheren*" OR "patient adheran*" OR "patient nonadheren*" OR "patient nonadheran*" OR "patient non-adheren*" OR "patient non-adheran*"OR "patient complian*" OR "patient noncomplian*" OR "patient non-complian*") AND (pharmac* OR drug* OR medication* OR medicine* OR "ART" OR "antiretroviral therap*" OR "anti-retroviral therap*" OR PReP OR "pre-exposure prophylaxis" OR "pre exposure prophylaxis" OR "preexposure prophylaxis"))) | Published Date: 20100101-20211231; English Language, Human, Male,  Academic Journals, USA |
| **Cochrane Library** | (MeSH descriptor: [HIV Infections] explode all trees OR MeSH descriptor: [Acquired Immunodeficiency Syndrome] explode all trees) AND (MeSH descriptor: [African Americans] explode all trees) AND (MeSH descriptor: [Medication Adherence] explode all trees AND MeSH descriptor: [Patient Compliance] explode all trees)  (Limitation: Searched only by subject heading) |  |
| **Embase** | **('patient compliance'**:ab,ti AND (pharmac*:ab,ti OR drug*:ab,ti OR medication*:ab,ti OR medicine*:ab,ti OR 'art':ab,ti OR 'antiretroviral therap*':ab,ti OR 'anti-retroviral therap*':ab,ti OR prep:ab,ti OR 'pre-exposure prophylaxis':ab,ti OR 'pre exposure prophylaxis':ab,ti OR 'preexposure prophylaxis':ab,ti) AND 'medication compliance'/exp OR ('medication adheren*':ab,ti OR 'medication complian*':ab,ti OR 'drug adheren*':ab,ti OR 'drug complian*':ab,ti OR 'medication noncomplian*':ab,ti OR 'medication non-complian*':ab,ti OR 'medication nonadheren*':ab,ti OR 'medication non-adheren*':ab,ti OR 'drug nonadheren*':ab,ti OR 'drug non-adheren*':ab,ti OR 'medicine adheren*':ab,ti OR 'medicine nonadheren*':ab,ti OR 'medicine non adheren*':ab,ti OR 'medicine non-adheren*':ab,ti OR 'medicine complian*':ab,ti OR 'medicine noncomplian*':ab,ti OR 'medicine non-complian*':ab,ti OR 'medicine non complian*':ab,ti)) AND (('human immunodeficiency virus infection'/exp OR 'acquired immune deficiency syndrome'/exp) OR 'human immunodeficiency virus infection':ab,ti OR 'acquired immune deficiency syndrome':ab,ti)) AND (('african american'/exp OR 'black person'/exp) OR ('african american*':ab,ti OR afroamerican*:ab,ti OR 'black person':ab,ti)) | [humans]/lim AND [english]/lim AND [2010-2021]/py |
| **IPA (Ovid)** | (("african american*" or afroamerican* or "black men" or "black man" or "black population" or "black people" or "black person*" or "black race" or "racially black" or "black homosexual*" or "black transsexual*" or "black trans-sexual*" or "black trans sexual*OR black bisexual*" or "black bi-sexual*" or "black bi sexual*" or "black gay*").mp) AND ("men who have sex with men" or "MSM" or "male homosexual*" or "homosexual male*").mp. OR ((men or man or male*).mp. ) AND (gay or gays or bisexual* or "bi-sexual*" or "bi sexual*" or queer or queers or "trans-sexual*" or "trans sexual*" or transsexual*).mp) AND ("AIDS" not ("communication aids" or "visual aids" or "teaching aids")).mp.) OR ("human immunodeficiency virus" or "HIV" or "Acquired Immunodeficiency Syndrome").mp) AND (("patient adheren*" or "patient adheran*" or "patient nonadheren*" or "patient nonadheran*" or "patient non-adheren*" or "patient non-adheran*OR patient complian*" or "patient noncomplian*" or "patient non-complian*").mp. AND (pharmac* or drug* or medication* or medicine* or "ART" or "antiretroviral therap*" or "anti-retroviral therap*" or PReP or "pre-exposure prophylaxis" or "pre exposure prophylaxis" or "preexposure prophylaxis").mp) | English language and human and last 11 years |
| **PubMed (NLM)** | (“African Americans”[Mesh] OR “African Continental Ancestry Group”[Mesh] OR “merica merican*” [tiab] OR “black men” [tiab] OR “black population”[tiab] OR “black man”[tiab] OR “black homosexual*”[tiab]) AND (“Homosexuality, Male”[Mesh] OR “Homosexuality”[Mesh] OR “Bisexuality”[Mesh] OR “Transsexualism”[Mesh] OR “Intersex Persons”[Mesh] OR “Transgender Persons”[Mesh] OR gays[tiab] OR “men who have sex with men” [tiab]) AND (HIV[Mesh]) OR “Acquired Immunodeficiency Syndrome”[Mesh] OR “Acquired Immunodeficiency Syndrome [tiab] OR “human immunodeficiency virus”[tiab] OR (“AIDS” [tiab] NOT (“communication aids” [tiab] OR “visual aids”[tiab] OR “teaching aids”[tiab])) OR “HIV”[tiab]) AND (“Medication Adherence”[Mesh] OR medication adheren*[tiab] OR “medication complian*”[tiab] OR “drug adheren*”[tiab] OR “drug complian*”[tiab] OR “medication noncomplian*”[tiab] OR “medication non-complian*”[tiab]OR “medication nonadheren*”[tiab] OR “medication non-adheren*”[tiab]) | Humans, English, Male, Publication Date 2010-> |
| **Web of Science Core Collection** | (TOPIC: "african american*" OR afroamerican* OR "black men" OR "black man" OR "black population" OR "black people" OR "black person*" OR "black race" OR "racially black" OR "black homosexual*" OR "black transsexual*" OR "black trans-sexual*" OR "black trans sexual*"OR "black bisexual*" OR "black bi-sexual*" OR "black bi sexual*" OR "black gay*" ) AND (TOPIC: "men who have sex with men" OR "MSM" OR "male homosexual*" OR "homosexual male*" OR ((men OR man OR male*) AND (gay OR gays OR bisexual* OR "bi-sexual*" OR "bi sexual*" OR queer OR queers OR "trans-sexual*" OR "trans sexual*" OR transsexual*)) AND (TOPIC: "human immunodeficiency virus" OR "HIV" OR "Acquired Immunodeficiency Syndrome" OR ("AIDS" NOT ("communication aids" OR "visual aids" OR "teaching aids")))  AND (TOPIC: "medication adheren*" OR "medication adheran*" OR "medication complian*" OR "medicine adheren*" OR "medicine adheran*" OR "medicine complian*" OR "drug adheren*" OR "drug adheran*" OR "drug complian*" OR "medication nonadheren*" OR "medication nonadheran*" OR "medication noncomplian*" OR "medication non-adheren*" OR "medication non-adheran*" OR "medication non-complian*" OR "medicine nonadheren*" OR "medicine nonadheran*" OR "medicine non-adheren*" OR "medicine non-adheran*" OR "medicine noncomplian*" OR "medicine non-complian*" OR "drug noncomplian*" OR "drug non-complian*" OR "drug nonadheren*" OR "drug nonadheran*" OR "drug non-adheren*" OR "drug non-adheran*" OR "drug noncomplian*" OR "drug non-complian*" OR (("patient adheren*" OR "patient adheran*" OR "patient nonadheren*" OR "patient nonadheran*" OR "patient non-adheren*" OR "patient non-adheran*"OR "patient complian*" OR "patient noncomplian*" OR "patient non-complian*") AND (pharmac* OR drug* OR medication* OR medicine* OR "ART" OR "antiretroviral therap*" OR "anti-retroviral therap*" OR PReP OR "pre-exposure prophylaxis" OR "pre exposure prophylaxis" OR "preexposure prophylaxis") | PubDate 2010->,  English, USA; Document Type: Article, Early Access, Review, Proceedings Paper, Meeting Abstract, Correction |
